# *Leishmania*-Host Interactions—An Epigenetic Paradigm

**DOI:** 10.3389/fimmu.2019.00492

**Published:** 2019-03-22

**Authors:** Farhat Afrin, Inbesat Khan, Hassan A. Hemeg

**Affiliations:** ^1^Department of Medical Laboratory Technology, Faculty of Applied Medical Sciences, Taibah University, Madina, Saudi Arabia; ^2^Rajiv Gandhi Technical University, Bhopal, India

**Keywords:** epigenetics, DNA methylation/demethylation, histone modification, non-coding RNA, leishmaniasis, host-pathogen Interactions, therapeutics, biomarkers

## Abstract

Leishmaniasis is one of the major neglected tropical diseases, for which no vaccines exist. Chemotherapy is hampered by limited efficacy coupled with development of resistance and other side effects. *Leishmania* parasites elude the host defensive mechanisms by modulating their surface proteins as well as dampening the host's immune responses. The parasites use the conventional RNA polymerases peculiarly under different environmental cues or pressures such as the host's milieu or the drugs. The mechanisms that restructure post-translational modifications are poorly understood but altered epigenetic histone modifications are believed to be instrumental in influencing the chromatin remodeling in the parasite. Interestingly, the parasite also modulates gene expression of the hosts, thereby hijacking or dampening the host immune response. Epigenetic factor such as DNA methylation of cytosine residues has been incriminated in silencing of macrophage-specific genes responsible for defense against these parasites. Although there is dearth of information regarding the epigenetic alterations-mediated pathogenesis in these parasites and the host, the unique epigenetic marks may represent targets for potential anti-leishmanial drug candidates. This review circumscribes the epigenetic changes during *Leishmania* infection, and the epigenetic modifications they enforce upon the host cells to ensure a safe haven. The non-coding micro RNAs as post-transcriptional regulators and correlates of wound healing and toll-like receptor signaling, as well as prognostic biomarkers of therapeutic failure and healing time are also explored. Finally, we highlight the recent advances on how the epigenetic perturbations may impact leishmaniasis vaccine development as biomarkers of safety and immunogenicity.

## Introduction

Epigenetics encompasses any process that changes gene expression and is inherited without amending the fundamental DNA sequence (1). These variations are highly dynamic which get altered on advent of any external stress or internal cues (2, 3). Epigenetics controls several cellular processes by switching genes on or off, thereby modulating gene expression. Epigenetics is also associated with various diseased states, wherein, it is shaped by host as well as pathogen selection pressures (4, 5). Recently, there is burgeoning interest in epigenetics landscapes during an infection, particularly alterations in DNA methylome, histone marks and non-coding (nc)RNA or micro (mi)RNA profiles. The epigenetic states result in erratic gene expression profiles of host cells, which are responsible for warding off microbial infections (6, 7).

*Leishmania* belongs to trypanosomatid family, being among the major neglected vector-borne tropical diseases, ranging in severity from self-healing but disfiguring and stigmatizing cutaneous lesions to disseminating muco-cutaneous and fatal visceral manifestations, depending on the species and host characteristics. Globally, 0.7–1.2 million new cases of cutaneous leishmaniasis (CL) occur every year; while for visceral leishmaniasis (VL), 200,000–400,000 new cases and 20,000–40,000 deaths are reported annually, with 95% of fatal cases occurring in only six countries, namely, India, Bangladesh, Sudan, Nepal, Ethiopia, and Brazil (8). The goal of World Health Organization is to eliminate this public health problem in South-east Asia region by 2020 (9).

*Leishmania* parasites have a digenetic life cycle that may be zoonotic or anthroponotic, depending upon the infecting parasite species. When an infected female sandfly (*Phlebotomus* or *Lutzomyia* species) takes a blood meal, the parasites cause dermal lesions as in CL or visceralize as in VL (10). The infection is amplified in the vector's gut with successive blood meals (11). Invasion of host macrophages by *Leishmania* triggers a multitude of signaling circuits to eliminate the pathogen. However, the parasite tries to subvert these defense mechanisms to create a safe haven for its survival. *Leishmania* secretes effector molecules to modulate host immune transcriptome resulting in alterations in the host epigenome, to alter cytokine and chemokine levels, their cross talks and downstream signaling hubs. This adversely affects the recruitment and activation of immune cells, respiratory burst and antigen presentation, leading to immune evasion (12).

**Figure 1:**
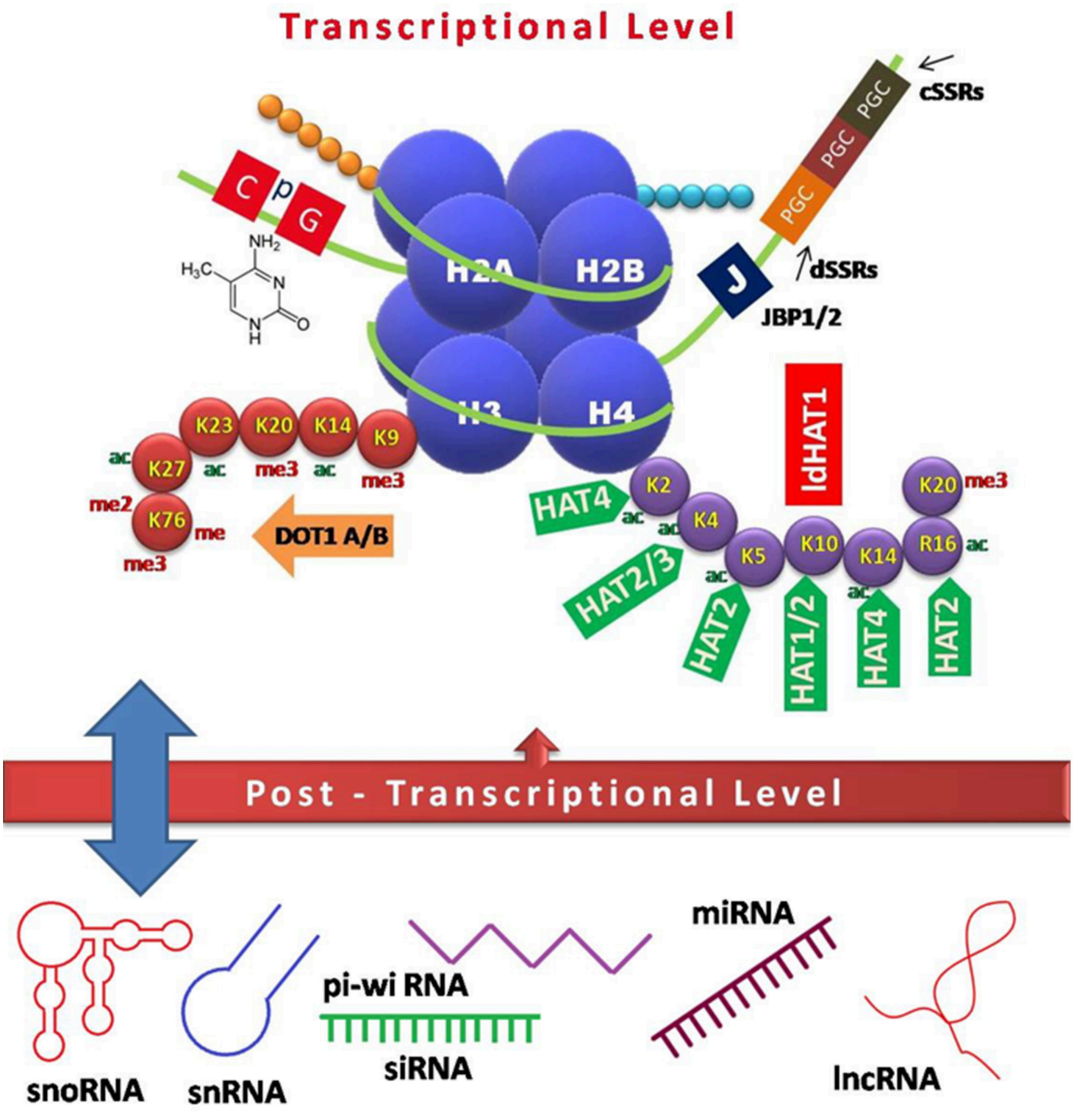
Schematic model of epigenetic regulation during *Leishmania* infection. Interplay of various factors involved in controlling gene expression at the transcriptional (DNA and histone modifications) and post-transcriptional level (non-coding RNAs) is depicted. CpG-rich regions of repressed genes are usually methylated, which in turn recruit chromatin modifiers to keep the genes in any of the three states, i.e., repressed, expressed, or poised. Heavily expressed genes show neither DNA methylation nor acetylated histones, while repressed genes tend to have both methylated DNA and histones, which inhibit the accessibility of polymerases, and other factors required for transcription. Base J, a DNA modification is crucial for transcriptional control in *Leishmania* species. Various non-coding RNAs arising mostly from UTRs act as regulatory elements in a feedback loop (13, 14). JBP, Base J binding protein; PGC, polycistronic gene cluster; cSSRs, convergent strand switch regions; dSSRs, divergent strand switch regions; DOT, disrupter of telomere; ac, acetylation; me, methylation; me2, dimethylation; me3, trimethylation; HAT, histone acetyl transferase; sno, small nucleolar; sn, small nuclear; pi-wi, piwi interacting; si, small interfering; mi, micro; lnc, long non-coding.

Though still at infancy, there is a recent surge of information on the epigenetic regulation during *Leishmania* infection. This review gives an update on *Leishmania* epigenetic landscapes and epigenome alterations imposed in the host for immune evasion (summarized in Figure 1 and Table 1). Further, evolving evidence on the probable downstream effects of epigenetic regulation such as targeting epigenetic machinery to reset the waning immune response, via vaccine, or drug development and prognostic markers are also discussed.

**Table 1:** *Leishmania* plasticity and *Leishmania-*induced host epigenetic alterations.

**Epigenetic regulator**	**Modification**	**Condition**	**Effect**	**Reference**
**EPIGENETIC REGULATION IN** ***LEISHMANIA***				
Base J	↑	*L. major*	Parasite survival	(15)
H2A.Z, H2B.V	↑	*L. major*	Parasite survival	(16)
HAT2	↑	*L. donovani*	Cyclins ↑, parasite survival	(17)
HAT3	↑	*L. donovani*	Parasite survival	(18)
HAT4	↑	*L. donovani*	Cyclins ↑, parasite survival	(19)
HDAC	↑	*L. infantum* logarithmic phase promastigotes versus intracellular amastigotes	Adaptation of amastigotes to phagolysosomal milieu	(20)
Sirtuin 2	↑	Amp B^R^ - *L. donovani*	ROS ↓, apoptosis ↓	(21)
***LEISHMANIA*****-INDUCED HOST EPIGENETIC ALTERATIONS**				
*FL1* methylation	↓	*L. braziliensis* infected møs from skin lesions	FLI gene expression	(22)
*IRAK2* DNA methylation at CpG sites	↑	*L. donovani* infected møs	IRAK2 mRNA ↓, NF-κB ↓, immune silencing	(23)
*LARS2* related CpG site methylation	↑	*L. donovani* infected møs	LARS mRNA ↓, mTORC1 ↓, 4E-BP1 ↑, parasite proliferation	(23)
*CDC42*EP3 methylation at CpG sites	↓	*L. donovani* infected møs	CDC42EP3 mRNA ↑, Progression of infection	(23)
HDAC4	↑	*L. donovani* infected møs	Phagolysosomal formation, amastigote survival	(23)
HDAC11	↑	Imipramine treated Sb^R^-*L. donovani* infected human møs	IL-12/IL-10 ratio ↑, parasite burden↓	(24)
HDAC1	↑	*L. amazonensis* infected møs	iNOS ↓, parasite survival	(25)
miRNA-294,−721	↑	*L. amazonensis* infected møs	Targets NOS-2, L-Arginine metabolism, NO ↓, parasite establishment	(26)
miRNA-210	↑	*L. major* infected møs	Activates hypoxia inducible factor-1α, parasite survival	(27), (28)
miRNA-129- 5p,−101c	↓	*L. major* infected møs	Autophagy ↑, infection↓	(28)
miRNA-25, - 26a,−140, - 155, let-7a	↑	*L. major* infected human møs	Corresponding chemokine targets ↓ (CCL5, CXCL10, CXCL11, CXCL12, CCL2)	(29)
miRNA-155	↑	*L. major* infected human DCs	PU.1 (SPI1) ↑, TGF-β signaling	(30)
let7a/b	↓	*L. major* infected human DCs, møs	Pro-inflammatory cytokines IL-12↓	(30)
miRNA-193b,−671	↑	Lesions from *L. braziliensis* infected patients	CD40, TNFR, inflammatory response, faster wound healing	(31)
miRNA-361- 3p	↑	Skin lesions from *L. braziliensis* infected patients	Therapeutic failure, healing time ↑, prognostic biomarker	(31)
miRNA-30A- 3p	↑	*L. donovani* infected THP-1, HMDMs	Autophagy ↓, promotes parasite survival	(32)
miRNA122	↓	*L. donovani* infected murine hepatocytes	Serum cholesterol ↓, maintains infection	(33)
miRNA-30c	↓	DBA-treated intramacrophagic *L. donovani* amastigotes	Inhibits proliferation and virulence	(32)
miRNA-151a	↓	DBA-treated intramacrophagic *L. donovani* amastigotes	Mitochondrial dysfunction	(32)
miRNA-6540	↓	*L. donovani* infected møs	Promotes intracellular parasite survival	(34)
miRNA- 3473f	↓	*L. donovani* infected møs	Autophagy ↓, role in pathogenesis	(34)
miRNA- 6973a	↑	*L. donovani* infected møs	IL-12 ↓, Th1 **→** Th2, parasite survival	(34)
miRNA-3620		*L. donovani* infected møs	Iron homeostasis genes, iron in cytoplasm, parasite survival	(34)
miRNA-3620,−6385	↑	*L. donovani* infected møs	Hypoxia inducing genes ↓, macrophage effector functions ↓, parasite survival	(34)
miRNA-763,−1264,−3473f	↓	*L. donovani* infected møs	ABC transporters ↑, drug efflux ↑, resistance	(34)
miRNA-21	↑	*L. donovani* infected human DCs	SMAD7 ↓, TGF-β signaling	(30)
miRNA- 146b-5p	↑	*L. donovani* infected human DCs	TRAF6 ↑, TGF-β signaling	(30)
let7a/b	↑	*L. donovani* infected human DCs, møs	Target pro-inflammatory genes, pro-inflammatory cytokines IL-12 ↓	(30)
miRNA-511	↑	*L. donovani* infected human DCs	TLR4 activation	(30)
miRNA-488i	↑	Sb^R^-*L. donovani* infected møs	MyD88 ↓, IL-10/IL-12 ↑ ratio, parasite number ↑	(35)
miRNA-34a	↓	*L. donovani* infected human møs	c-myc ↑, M2 mø activation, attenuates parasite survival	(36)
miRNA-155	↑	*L. infantum* infected J774 møs	Susceptibility to Sb ↓	(25)
miRNA-191,−374	↑	*L. infantum* infected dog PBMCs	Parasite load ↑	(37)
miRNA-150	↓	*L. infantum* infected dog PBMCs	Parasite load ↑	(37)

*HDAC, Histone deacetylase; IRAK2, interleukin-1 receptor associated kinase 2; LARS, leucyl-tRNA synthetase; Amp, B^R^ Amphotericin B resistant; møs, macrophages; HMDMs, Human monocyte derived macrophages; DBA, dibenzalacetone; ABC, ATP-binding cassette; DCs, Dendritic cells; I, inhibitor; Sb^R^, Antimony resistant; Sirtuins, Silent Information Regulator*.

## Epigenetic Changes in *Leishmania* Parasites

The diverse clinical manifestations of leishmaniasis may be attributed to varying genomic makeup (size, GC content, coding genes, pseudogenes, retrotransposons) of the causative *Leishmania* species (38). To promote their survival in the host environment, a huge array of epigenetic factors is speculated to interplay in these parasites (39).

### DNA Modification

Glycosylated thymine, termed “Base J” or β-D-glucosylhydroxymethyluracil, is present in the telomeric repeat sequence (GGGTTA) of *Leishmania* (40). J replaces ~1% of T in nuclear DNA and the modified T residue has been implicated in transcriptional regulation and termination. Absence of J is lethal to *Leishmania*, due to massive read through of transcriptional termination sites (41). However, repression of specific genes has now been identified as an essential role of Base J (15). A thymine base modification, 5-hydroxymethyluracil has recently been mapped to *Leishmania* genome, but its epigenetic role is yet to be elucidated (42).

### Histone Alterations

Acetylation of histone H3 in telomeric divergent strand switch regions, has been reported in *L. major* promastigotes (43) resulting in chromatin state, with restriction of protein coding genes. Acetylation levels are higher in rapidly growing cells compared to stationary phase cells. Epigenetic marks such as H3K9me3, H3K14ac, H3K23ac, and H3K27ac have also been reported in promoter region of rRNA genes of *L. major*, favoring transcriptional activation of rRNA genes while H4K20me3 in the coding region is related to transcriptional silencing (44). H3K9me3 is also linked to heterochromatin formation. Histone variants such as H2A.Z and H2B.V have been identified as essential for *L. major* survival (16).

In *L. donovani*, histone acetyltransferase (HAT)4 acetylates H4K14, favoring maintenance of euchromatin state (19). H4K4 acetylation by HAT3 (18) and, HAT2-dependant H4K10 acetylation of promoters in *L. donovani* has been linked with parasite survival (17).

Epigenetic tags are differentially regulated in the promastigote and amastigote stage. Some of the histone deacetylases (HDAC) are preferentially upregulated in *L. infantum* logarithmic phase promastigotes over intracellular amastigotes, making the amastigotes better able to adapt to intraphagolysosomal environment (20). Sirtuins, NAD-dependent HDAC have been implicated in parasite survival by inhibiting apoptosis (45), and sirtinol, sirtuin inhibitor, selectively induced apoptosis in *L. infantum* axenic amastigotes but not the promastigotes (46).

### Non-coding RNAs

ncRNAs, non-coding sequences of about 22 nucleotides, act as post-transcriptional regulators of RNA encoding proteins (47). A special class of nc RNAs found exclusively in amastigotes of *L. infantum* and *L. donovani*, is particularly important for intra-macrophage parasite survival (48). In *L. tarentole*, ncRNA similar to guide RNA encoded by maxicircles and minicircles has been identified (49). Significant differences in nc RNA repertoire among different *Leishmania* species and stages has been reported (50).

## *Leishmania*-Induced Host Epigenetic Alterations

Epigenetic mechanisms regulate the interplay of host-pathogen interactions. Although information on epigenetic manipulation of hosts by *Leishmania* is scarce, the pathogen employs a number of stratagems to manipulate the host epigenome, thereby hijacking its cellular soldiers (23, 51, 52). Genetic heterogeneity among different *Leishmania species* causes altered gene expression in response to environmental conditions in the host, resulting in varied epigenetic mechanisms.

### DNA Modifications in Host

*L. donovani* has been reported to elicit epigenetic modifications in host macrophages, permanently down-modulating the innate immune defenses (23). These altered epigenetic tags comprise of cytosine methylation at CpG sites of macrophage DNA, upon infection, causing alteration in genes implicated in JAK/STAT, calcium, MAPK, notch, and mTOR signaling pathway as well as in cell adhesion involving integrin β1 and changes in host oxidative phosphorylation. *Leishmania*-driven epigenomic changes in host macrophages deactivate its innate immune defensive machinery, thereby promoting pathogen survival, and replication.

Epigenetic modification promotes self-healing in CL. Epigenetic repression of wound healing gene, Friend leukemia virus integration 1 (*FLI1*) via increased methylation of CpG islands in its promoter region, has been found to correlate with up-modulation of pro-fibrotic genes such as collagen type I alpha 1 (*Col1*α*1*) and alpha 2 (*Col1*α*2*) and, conversely, with down-regulation of matrix metalloproteinase 1 (*MMP1*) gene, resulting in resolution of lesions caused by *L. braziliensis* (53, 54). *MMP1* cleaves type I collagens to loosen keratinocytes-dermal matrix contact, favoring re-epithelialization or tissue repair. Homocystine-dependent stimulation of IL-6 has been further implicated in epigenetic DNA methylation of CpG-rich promoter of lysyloxidase (*LOX*) gene, a cross-linker of collagen and elastin, also rendering it transcriptionally inactive (55, 56). These epigenetic regulations of gene expression depend upon the infecting *Leishmania* species. Contrary to these reports, a recent study showed that increased *FLI1* promoter methylation did not translate into low *FLI1* gene expression (22).

### Histone Modifications in Host

*L. amazonensis* induces HDAC in infected macrophages, contributing to down regulation of inducible nitric oxide synthase (iNOS) and subsequent parasite survival (25).

### ncRNA Induced Gene Silencing

*Leishmania* infection targets cellular miRNA repertoire and the differential miRNA expression is dependent on infecting species (37). A plethora of studies indicate miRNAs as key regulators of disease phenotype in *Leishmania*-infected cells (27, 30, 32–34). miRNA-30A-3p mediates survival of intracellular *L. donovani* and intervention targeting the miRNA resulted in significant reduction in parasite burden by restoring host autophagic machinery (32). miRNA-3620 was found to regulate iron homeostasis and hypoxia in *L. donovani* infected macrophages while miRNA-3473f was linked with autophagy inhibition (34). Drug resistance due to over expression of efflux pumps such as ABC transporters has also been linked with downregulation of miRNA-763,−1264, and−3473f (34). *L. donovani* infection causes hypoxic environment within the macrophages by activating hypoxia inducible factor-1α, that in turn up regulates miRNA-210, while down regulating NF-κB mediated pro-inflammatory immune responses, to establish a safe niche for its survival (57).

*Leishmania* establishes and survives in the host by manipulating its ncRNA network, which includes transcriptional arrest of the major protein coding genes in macrophages (58), downregulating 7SL RNA in SRP complex, knockdown of selected ncRNAs in their host cells by inducing degradation of a specific RNA Pol III transcription factor subunit TFIIIC110 in M2 macrophages (59). *Leishmania* surface glycoprotein, gp63 and surface glycolipid, LPG have been reported to down modulate ncRNAs in M2 macrophages, thereby promoting infection (60).

Recently, down modulation of 19 miRNAs in *L. donovani* infected macrophages has been reported (36). The miRNA gene repression correlated with upregulation of host transcription factor, c-myc upon infection, a marker of M2 macrophages, which could possibly be another virulence factor. The expression of c-myc in turn is regulated by several miRNAs, primarily miRNA-34a, which is reciprocally down modulated in *Leishmania-*infected cells.

miRNA-361-3p and−140-3p have been reported to be more expressed in skin lesions caused by *L. braziliensis* in localized cutaneous leishmaniasis (LCL) (61). While miR-193b and−671 have been correlated with faster wound healing in *L. braziliensis* infected patients (31). Autophagy in intramacrophagic *L. major* has been correlated with miRNA-101c,−129-5p and via inhibiting miRNA-210 (28).

## Potential Downstream Effects of Epigenetic Regulation During *Leishmania* Infection

### Epigenetic Reprogramming of Innate Immune Cells

Recent reports shed light on epigenetic reprogramming in monocytes and macrophages via histone trimethylation at H3K4 for innate immune memory or trained immunity (62, 63). Natural killer (NK) cells have also been reported to differentiate into memory NK cells with distinct epigenetic profile (64). However, the epigenetic signatures of innate immune cells during *Leishmania* infection are limited (65).

### Epigenetic Tuning of Cell Signaling Hubs

Epigenetic reprograming at cytokine gene loci is reported to influence its gene expression. A growing body of data suggests that differential cytokine microenvironment modulates T helper (Th) cell polarization, macrophage phenotype differentiation and cytokine-inflammasome crosstalk for optimal immune response (65). Signal transducers and activators of transcription (STAT)-4 and−6 have also been reported to play antagonistic roles in epigenetic tuning for Th cell differentiation (66). The epigenetic marks orchestrating gene regulation in Th cell differentiation (67) and M1/M2 macrophage polarization have been extensively reviewed (68).

Differential expression of miRNAs has been reported to induce T cell differentiation during VL. While miRNA-744 suppresses TGF-β expression and subsequently Treg cell differentiation, miRNA-1272 and−155 downregulate IL-4/IL-13 signaling to mitigate Th2 response during active infection (69). Antimony-resistant *L. donovani* has been reported to activate miRNA-466 inhibitor to degrade host MyD88 and regulate IL-10/IL-12 axis and establish successful infection (35). Chemokine and chemokine receptor gene expression also contribute to immunopathogenesis of leishmaniasis (70). But the effect of *Leishmania*–induced epigenetic alterations in regulation of chemokine genes has not been much explored (29).

miRNAs are also known to be involved in activation of monocytes through toll-like receptor (TLR) signaling (31). Following infection with *L. major* and *L. donovani*, miRNA expression was down modulated through MAP kinase, JAK-STAT, and TGF-β signaling pathways (31). H3K27 has been found to suppress toll-interacting protein that negatively regulates TLR, thereby promoting TLR-mediated inflammatory cytokine production, and activation of innate immune response against the invading pathogens (65).

## Exploiting Epigenetics

*Leishmania* have evolved stratagems to neutralize macrophage defensive arsenals, the very heart of immune system's defensive machinery, resulting in replication of parasites within phagolysosomal vacuoles of infected macrophages. Unfolding the epigenetic signatures of host-pathogen interactions would help in development of effective drug targets to modulate host immune system and ameliorate the pathogenesis of infection. Some epigenetic marks may serve as putative vaccine candidates. The epigenetic biomarkers may also complement the current diagnostic assays.

### Vaccines

An essential hallmark of vaccination is to generate antigen-specific memory T cells for induction of sufficient immune response to protect against re-infection. Epigenetic modifications have been reported to contribute toward memory T cell induction (71, 72). Recombinant histone H1 has been shown to elicit protection in outbred vervet monkeys against CL (73) while histones H2A-2B-3-4 cocktail induced protective immunity against *L. donovani* challenge in hamsters (74). Sirtuins have been used as vaccine candidate against *L. donovani* infected hamsters with induction of Th1 immune response (75). Recently, miRNA-21 has been shown to negatively correlate with IL-12 production and priming of protective Th1 response, suggesting declining levels of miRNA-21 as a potential biomarker of safety and immunogenicity in anti-leishmanial vaccines (76). Therapeutic vaccines may be developed to target miRNA-135 and−126 that bias the Th2 response toward protective Th1 type (69).

### Epigenomic-Therapeutics

Despite an array of chemotherapeutic arsenal, mostly targeting the parasites directly, treatment failure, and drug resistance are looming large (77). This has been partly attributed to epigenetics-driven evolution of drug resistant phenotypes to override drug pressure (78). Host-directed epigenetic reprogramming may be refractory to resistance and hence offer hope in this regard (79).

Computer-aided drug repurposing for epigenetic targets is revolutionizing drug discovery (80). DNA methylation, particularly of virulence-associated genes, suggests DNA methyl transferases as potential therapeutic targets. An inverse correlation between *FLI1* gene expression and *MMP1* in cutaneous lesions has also been observed, suggesting *MMP1* as a potential therapeutic target in severe forms of leishmaniasis (22). *FLI1* and *LOX* have also been implicated as potent drug targets in *L. braziliensis* infection (54, 56).

The enzymes effecting histone post-translational modifications, particularly those containing epigenetic reader modules, bromodomains could also be putative therapeutic targets. Sirtuins of *L. donovani* have been validated as drug targets (81). Crystal structure of *L. infantum* Sir2 has been elucidated with implications for drug design (82). Sir2 has been suggested as a resistance marker for VL (21). Phenotypic screening of compound libraries against *Leishmania* has helped in identification of bisnaphthalimidopropyl derivatives as sirtuin inhibitors (83). Imipramine has been found to mediate antileishmanial effect in antimony-resistant *Leishmania*-infected macrophages via targeting HDAC11, resulting in transcriptional inactivation of IL-10 production (24). KH-TFMDI, a novel sirtuin inhibitor, targets HDAC to promote apoptosis-like cell death in *L. amazonensis* promastigotes as well as intracellular amastigotes (84). However, none of the clinically approved HDAC inhibitors are effective against *L. amazonensis* (85).

Studies have deciphered role of miRNA-294 and−721 in *Leishmania* survival via subversion of macrophage nitric oxide production and hence these may be putative therapeutic targets (26, 32). Recent reports of *L. donovani* hijacking the host's transcription factor, c-myc and reduction of intramacrophagic parasite burden upon c-myc silencing or inhibition, with consequent miRNA upregulation, implicate c-myc as a potential therapeutic target (36). Epigenetic targets such as miRNAs screened in *L. donovani*-infected macrophages upon treatment with antileishmanial trans-dibenzalacetone, revealed an imbalance between apoptosis and autophagy (86).

### Epigenetic Biomarkers

*Leishmani*a-induced changes in hosts' epigenome may help to predict the clinical outcome of infection and hence complement the existing diagnostics. The state of knowledge regarding epigenetic biomarkers in leishmaniasis is limited. A recent study showed potential of miRNA-361-3p as a prognostic biomarker in CL caused by *L. braziliensis* (61). miRNA-361-3p expression was upregulated in patients with therapeutic failure to pentavalent antimony and hence required more healing times. miRNA-193b and−671 have also been speculated to be prospective biosignatures for prognosis of LCL but require further validation (31).

## Concluding Remarks

The epigenetic mechanisms work in alliance with each other to regulate life cycle of *Leishmania* parasites and ensure their survival. Pathogens are also capable of eluding cellular defensive machinery by changing the epigenetic states of host gene expression, thereby dampening their immune response. A snapshot of epigenetic imprinting of relevant genes in Th cell polarization, and memory T cell differentiation with triggering of innate immune cell populations may provide a basis for development of improved leishmaniasis vaccines.

Targeting the epigenetic marks could result in drug design with less likelihood of development of resistance, thus extending the pipeline toward disease elimination. Whether *Leishmania* parasites tailor the epigenetic mechanisms of their vector sandfly to favor their colonization remain to be elucidated. The impact of these pathogens on vector epigenetics could pave a way for development of transmission blocking vaccines. This review may assist to expand our knowledge of epigenetic influences upon host-parasite interplay, and open the doors to investigate epigenetic targets for rapid diagnostics or therapeutic interventions.

## Author Contributions

FA: conceptualization, reviewing the studies, writing the mini-review, and critical editing; IK: conceptualization, writing original draft, editing; HH: reviewing the draft; critical editing. All the authors are accountable for all aspects of the review and gave final approval of the version to be published.

### Conflict of Interest Statement

The authors declare that the research was conducted in the absence of any commercial or financial relationships that could be construed as a potential conflict of interest.
